# BODY WEIGHT CHANGE IN OLDER PEOPLE DURING THE COVID-19 PANDEMIC: RESULTS FROM THE BERLIN AGING STUDY II (BASE-II)

**DOI:** 10.1093/geroni/igae098.2324

**Published:** 2024-12-31

**Authors:** Valentin Vetter, Johanna Drewelies, Sandra Düzel, Jan Homann, Lars Bertram, Vera Regitz-Zagrosek, Denis Gerstorf, Ilja Demuth

**Affiliations:** Charité - Universitätsmedizin Berlin, Berlin, Berlin, Germany; Max Planck Institute for Human Development, Berlin, Berlin, Germany; Charité - Universitätsmedizin Berlin, Berlin, Berlin, Germany; University of Münster, Münster, Nordrhein-Westfalen, Germany; University of Lübeck, Lübeck, Schleswig-Holstein, Germany; Charité - Universitätsmedizin Berlin, Berlin, Berlin, Germany; Humboldt University Berlin, Berlin, Berlin, Germany; Charité - Universitätsmedizin Berlin, Berlin, Berlin, Germany

## Abstract

Low body weight and especially unwanted weight loss were shown to be associated with higher mortality in older adults. Change in body weight was reported to be one of the unintended side effects of lockdown measures that were put in place to combat the COVID-19 pandemic. In this study, we first describe weight change in 472 participants (mean age: 67.5 years, 48% women) of the Berlin Aging Study-II (BASE-II) that was collected at four timepoints over an average total follow-up time of 10 years. In a second step we examine differences in weight change between groups defined by socio-economic, cognitive, and psychosocial variables as well as morbidity burden, biological aging markers (epigenetic clocks, telomere length), and frailty. During the follow-up period, which includes the first six months of the pandemic, women and men lost on average 0.87% and 0.5% of their body weight per year. A statistically significantly higher rate of weight loss was detected for men with low positive affect, accelerated epigenetic age (7-CpG clock), diagnosed metabolic syndrome as well as a more masculine gender score (all p< 0.05). Although the literature generally suggests an increase in body weight during the pandemic, older participants of the BASE-II sample lost 2.5-times (women) and 2-times (men) more weight than expected in this age group. Our explorative analysis of additional variables assessed during an examination directly before the pandemic’s onset could inform future studies that aim at a more in depth understanding of the matter.

